# Process monitoring for quality — A multiple classifier system for highly unbalanced data

**DOI:** 10.1016/j.heliyon.2021.e08123

**Published:** 2021-10-06

**Authors:** Carlos A. Escobar, Daniela Macias, Ruben Morales-Menendez

**Affiliations:** a Global Research & Development, General Motors, Warren, MI, USA; b Tecnolo´gico de Monterrey, Monterrey, NL, Mexico

**Keywords:** Defect detection, Binary classification, Manufacturing, Ensemble learning, Quality control, Machine learning, Unbalanced data, Multiple classifier system, Meta-learning

## Abstract

In *big data-*based analyses, because of hyper-dimensional feature spaces, there has been no previous distinction between *machine learning algorithms* (*MLAs*). Therefore, multiple diverse algorithms should be included in the analysis to develop an adequate model for detecting/recognizing patterns exhibited by classes. If multiple classifiers are developed, the next natural step is to determine whether the prediction benchmark set by the top performer can be improved by combining them. In this context, *multiple classifier systems* (*MCSs*) are powerful solutions for difficult pattern recognition problems because they usually outperform the best individual classifier, and their diversity tends to improve resilience and robustness to high-dimensional and noisy data. To design an *MCS*, an appropriate fusion method is required to optimally combine the individual classifiers and determine the final decision. *Process monitoring for quality* is a *Quality 4.0* initiative aimed at defect detection via binary classification. Because most mature organizations have merged traditional quality philosophies, their processes generate only a few defects per million of opportunities. Therefore, manufacturing data sets for binary classification of quality tends to be highly/ultra-unbalanced. Detecting these rare quality events is one of the most relevant intellectual challenges posed to the fourth industrial revolution, *Industry 4.0* (*I 4.0*). A new *MCS* aimed at analyzing these data structures is presented. It is based on eight well-known *MLAs*, an *ad hoc* fitness function, and a novel meta-learning algorithm. For predicting the final quality class, this algorithm considers the prediction from a set of classifiers as input and determines which classifiers are reliable and which are not. Finally, to demonstrate the superiority of the *MLAs* over extensively used fusion rules, multiple publicly available data sets are analyzed.

## 1 Introduction

The *fourth industrial revolution* (*I 4.0*) is changing the way we work, live, and interact with one another. It is founded on new technologies such as *industrial Big Data*, *Industrial Inter-net of Things* (*IIoT*), and *Artificial Intelligence* (*AI*), and it fuses physical systems with virtual ones, thus enabling a smart and connected world. *I 4.0* is impacting all scientific disciplines, industries, and economies. A simulation study demonstrated that by 2030, 70% of companies might adopt at least one type of *AI* technology and potentially generate an additional economic activity of $13 trillion USD [1]. However, new business models such as *Amazon*, *Facebook*, and *Uber* have recently emerged and are propelling the *I 4.0*. Manufacturing science has been innovating, advancing, and evolving after the beginning of the first industrial revolution [2]. At present, manufacturing is the driving economic force of the most advanced countries [3]. Top-ranking nations in overall manufacturing environment [4] have the highest *gross domestic product* (*GDP*) [5]. Although not a trivial task, the driving technologies in *I 4.0* have the capacity to further the *state-of-the-art* of manufacturing science.

**Figure 1:**
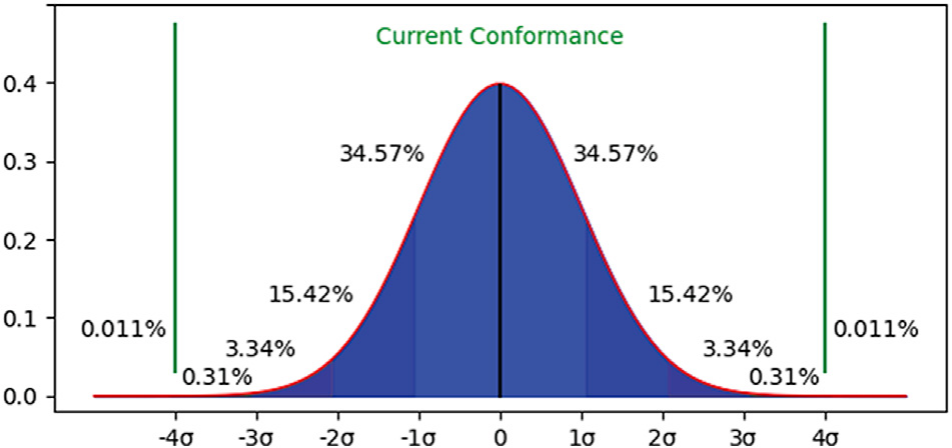
Current conformance rate across manufacturing companies.

From the perspective of manufacturing quality, most mature organizations have merged traditional quality control methods to create high-conformance production environments. Process monitoring charts have been developed to improve the process capability index at the industrial benchmark of *sigma level four* [6, 7]. This sigma level generates 6,210 *defects per million of opportunities* (*DPMOs*) [8, 9]. Detecting these defects to move manufacturing processes to the next *sigma level* is one of the primary intellectual challenges posed to *AI* [10] (see Figure 1).

**Figure 2:**
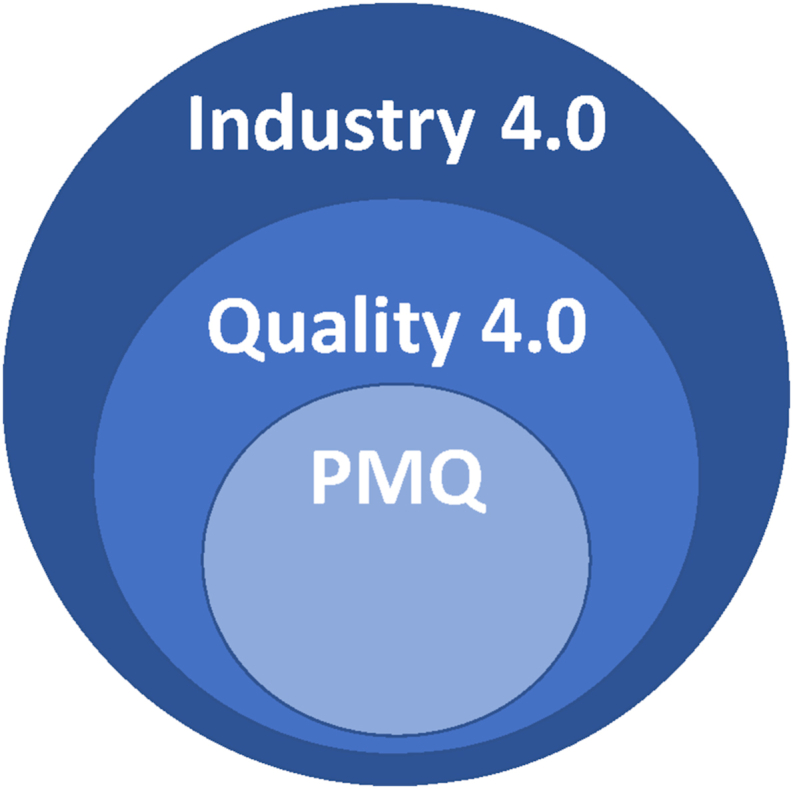
*PMQ* in the context of Industry 4.0.

**Figure 3:**
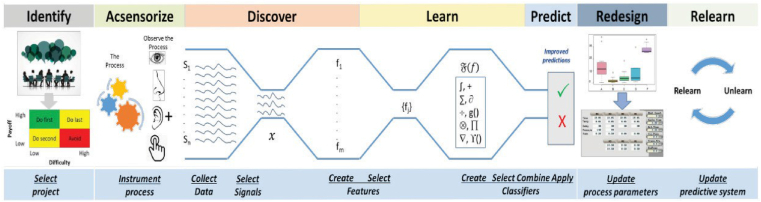
*PMQ* problem solving strategy.

*Process monitoring for quality* (*PMQ*) is a *Quality 4.0*^1^ initiative founded on *AI* (Figure 2). Detecting rare quality events or few *DPMOs* generated by a typical manufacturing process is one of the primary goals. Defect detection is formulated as a binary classification problem (good or defective) [11]. *PMQ* has evolved the traditional quality problem solving strategies (*PDCA*, *DMAIC*, *IDDOV* [12]) into a seven-step *approach* —(*Identify, Acsensorize, Discover, Learn, Predict, Redesign, Relearn: IADLPR*^2^) — to effectively solve pattern classification problems and to guide (Figure 3) [12,13]. An approach to solve the *predict step* (or letter *P*) of the problem solving strategy is presented.

From the *ML* perspective, manufacturing-derived data sets for binary classification of quality tend to be highly/ultra-unbalanced (minority class count <1%). Therefore, a learning strategy (e.g., hyper-parameter tuning, classification threshold definition, and misclassification cost) must be developed to address this situation. Owing to the limited amount of information available of the minority class, it is extremely complicated for an *MLA* to capture this pattern.

In *big data*-based analyses, because of hyper-dimensional feature spaces, the data structure is not known in advance. There is, therefore, no *a priori* distinction between *MLAs* [14]. Multiple *MLAs* should be included in the analysis to develop an adequate model for the pattern exhibited by the classes. Moreover, based on empirical evidence, diversity tends to improve the prediction performance, resilience, and robustness of high-dimensional and noisy data [15].

If multiple classifiers are developed, the next natural step is to combine them to optimize prediction. The most common fusion rules are majority, simple majority, and unanimous voting [15]. Recently, a majority voting, *Multiple Classifier System* (*MCS*) concerning biological activities was developed [16]; this predictive system outperformed individual classifiers by addressing the over-fitting problem. However, in an *MLA*, the concept of drift [17, 18] addresses the fact that the statistical distributions of classes change over time in an unforeseen manner. This poses important technical and practical challenges because a stationary relationship is assumed between features and classes. Assumption is rarely held by manufacturing systems [13]. Moreover, the pitfall of using predefined classification rules was acknowledged in Ref. [19].

If extensively used or existing static *fusion rules* rarely generalize/sustain in manufacturing, the research question addressed in this study is: which *fusion rule* should be used to optimize the detection of rare events? A novel *meta-learning algorithm* [20] was developed to answer this question.

The process of developing an *MCS* is divided into two optimization goals: (1) coverage optimization, an approach aimed at increasing the hyper-dimensional space covered by a set of mutually complementary classifiers, and (2) decision optimization, a *meta-learning algorithm* aimed at designing an appropriate decision combination scheme (i.e., fuser) over a set of previously trained classifiers [21, 22].

A prediction optimization approach, *PMQ-O*, is presented. It is an effective strategy aimed at developing an *MCS* with the capacity to analyze highly/ultra-unbalanced data. The proposed approach is based on the following: (1) a list of eight diverse *MLAs*, (2) an *ad hoc* fitness function, and (3) a new *meta-learning algorithm* that searches for an optimal solution. These three components address the two optimization goals. As demonstrated by multiple empirical studies, *PMQ-O* is a step forward in moving manufacturing processes to *the next sigma level*.

The rest of this study is organized as follows. A general scheme of *PMQ* is presented in Section 2. A brief theoretical background of this study is presented in Section 3. An optimizer is presented in Section 4. Empirical studies are outlined in Section 5, wherein a virtual case study is presented, aimed at explaining the optimizer in a step-by-step manner, followed by the analysis of six real data sets. *Industry 4.0* technologies and sustainability are mentioned in Section 6. Finally, Section 7 concludes this paper. Table 1 summarizes the acronym definitions.

**Table 1:** Acronym definitions.

Acronym	Definition
*AI*	Artificial Intelligence
*ANN*	Artificial Neural Networks
*BM*	Big Models
*CM*	Confusion Matrix
*DPMO*	Defects Per Million of Opportunities
*FN*	False Negative
*FP*	False Positive
*GDP*	Gross Domestic Product
*IADLPR*^2^	Identify, Acsensorize, Discover, Learn, Predict, Redesign, Relearn
*IIoT*	Industrial Internet of Things
*I4.0*	Industry 4.0
*KNN*	K-Nearest Neighbors
*MCS*	Multiple Classifier System
*LR*	Linear Regression
*ML*	Machine Learning
*MLA*	Machine Learning Algorithm
*MPCD*	Maximum Probability of Correct Decision
*NB*	Naive Bayes
*PMQ*	Process Monitoring for Quality
*PMQ-O*	Prediction Optimization approach
*RF*	Random Forest
*RBF*	Radial Basis Fusion
*QMC*	Quality Monitoring and Control
*SVM*	Support Vector Machine
*TN*	True Negative
*TP*	True Positive

## 2 Process monitoring for quality

In *PMQ*, the *Big Models* (*BM*) learning paradigm [23] is applied to process data to develop a classifier aimed at defect detection. Using the notation of eqn. (1), a positive label refers to a defective item, whereas a negative one refers to a good quality item.

(1) $$Quality=\left\{ \begin{array}{ll} 1 & \text{if ​}i^{th}\;\text{item ​is ​}defective\left(+\right)\\ 0 & \text{f ​}i^{th}\;\text{item ​is ​}good\left(-\right) \end{array}\right.$$

The predictive performance of a classifier is summarized using a *confusion matrix* (*CM)* (see Table 2).

**Table 2:** Confusion matrix.

	Predicted good	Predicted bad
Good item	True Negative (*TN*)	False Positive (*FP*)
Defective item	False Negative (*FN*)	True Positive (*TP*)

Because predictions are performed under uncertainty, a classifier can commit FP (type-I, α) and FN (type-II, β) errors [24]. In the context of binary classification of quality, an *FP* error occurs when a classifier labels a good item as a defective one, whereas an *FN* error occurs when a defective item is labeled as good. Errors are computed by the following equations:

(2) $$\alpha =\frac{FP}{FP+TN}$$

(3) $$\beta =\frac{FN}{FN+TP}$$

**Figure 4:**
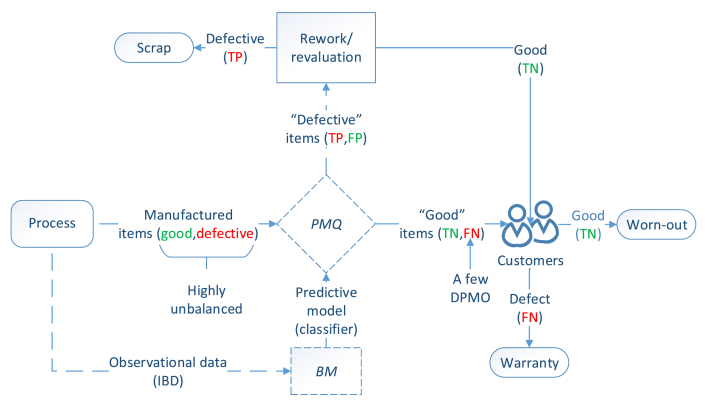
*PMQ* scheme.

Figure 4 shows a typical high-conformance process controlled by *PMQ* where observational (i.e., empirical) data are used to train a classifier following the *BM* learning paradigm [23]. From a theoretical perspective, applying *ML* to detect the minority class is the primary challenge, whereas from practical/business perspectives, the goal is to develop defect-free processes [10]. Because an *FN* can be reevaluated at a minimum cost and continue in the value-adding process, the classifier must exhibit a predominantly high detection ability (β ≈ 0) and the smallest possible α error.

*PMQ* uses a seven-step problem solving strategy to guide innovation. *IADLPR*^2^ is developed based on theory and our knowledge of complex manufacturing systems. As per empirical results, this strategy increases the likelihood of success by addressing the primary challenges of manufacturing systems [ 11,^13^, ^23^]. Table 3 lists the primary goals for each step of this strategy.

**Table 3:** Seven steps and goals.

Step	Goal
Identify	to develop a prioritized portfolio of projects with a high business impact and likelihood of success.
Acsensorize	to observe the process and generate the raw empirical data to monitor the system to create features
Discover Learn	to develop the classifier using the *Big Models* learning paradigm
Predict	to optimize prediction (this paper goal)
Redesign	to derive engineering knowledge from the data mining results
Relearn	to develop a relearning strategy for classifier to learn new statistical distributions classes

## 3 Theoretical background

The overall research goal of *AI* is to create technologies that augment human intelligence or take over risky jobs not appropriate for humans. *ML*, robotics, computer vision, natural language processing, and expert systems are the common *AI* areas [25].

*ML* serves as a tool for information extraction, data pattern recognition, and prediction. Although it becomes increasingly challenging to build first-principle models in these increasingly complex processes, data-driven process modeling, monitoring, prognosis, and control have recently received considerable attention. Manufacturing must be reimagined in the new era of data science and information technology.

The primary goal of data mining is to extract beneficial details and transfer them to practical knowledge to improve process decision support. Multiple tasks require pattern recognition, reasoning, and decision-making under complex conditions. Furthermore, they often deal with *ill-defined problems*, noisy data, model uncertainties, combinatorially large search spaces, non-linearities, and the requirement for speedy solutions. Such features are reported in many issues in the process industry—in synthesis, design, control, scheduling, optimization, and risk management.

There are three different types of *MLA*: *unsupervised methods*, *supervised learning methods* (*SVMs*), and *reinforcement learning methods*. Supervised and unsupervised learning methods account for 80%–90% of all industrial applications, [26]. With *I 4.0*, *IIoT*, and *cloud computing*, *MLA*-based *quality monitoring and control* (*QMC*) has successfully resurfaced in various industrial domains. For example, real-time data acquisition systems enable an *MLA* application to accurately assess the quality of a manufacturing process.

A feedback (*adaptive and self-updating*) system with sufficient data is essential to ensure the effectiveness of an *MLA* over time, which enables the real-time improvement in quality inspection. Furthermore, an *MLA* can be used to devise complex models and algorithms that can lend themselves to prediction. These algorithms allow for people to produce reliable, repeat-able decisions and uncover hidden insights by learning from historical relationships and trends in the data.

An algorithm integrating feedback and an adaptable process that is analyzed over time in the long run to improve *SVM*-based systems was proposed in [27]. The adaptive approach considerably improved the feedback process and effectively enhanced the system accuracy and reliability over time. The framework to pre-process the input data for *SVM*-based decision-making algorithms was applicable for *MLAs*, e.g., cost-effective *SVM*-based automated *QMC*. It incorporated inspection-related expenses (warranty cost, rework cost, inspection cost) and error types (type *I* and *II* errors) in the algorithm [28]. In this proposal, the quality check reflected the company priorities regarding cost-saving policy versus traditional *SVM* methods that employ the lowest inspection error rate criterion for classification. Essential features were considered the ability to learn from and make predictions based on defective parts.

In addition to these *SVM*-based applications, there are many more that combine two *MLAs* to generalize the practical approach: *MCSs*. To develop an *MCS*, three questions must be answered: (1) which classifiers should be included?, (2) which fitness function should be optimized?, and (3) how should the predictions (labels) of the classifiers be combined?

An overview of *aggregation methods* and *meta-learning* in *ML* is presented. Thereafter, a brief theoretical review is provided to answer the first two questions, and the third one is answered in Section 4.

### 3.1 Aggregation methods

For an *ensemble learning* approach (voting by several classifiers), a group of homogeneous classifiers is called an *ensemble* [29], e.g., bagging and boosting [29, 30, 31]. However, a group of heterogeneous classifiers is called an *MCS* or *non-ensemble* [32, 33]. In the context of ideas expressed in this study, an *MCS* is a predictive approach that may include a com bination of classifiers with *ensembles*.

*MCSs* are a powerful solution to difficult pattern recognition problems because they usually outperform the best individual classifiers [34]. This improvement has been analytically proven under certain conditions (e.g., majority voting by a group of independent classifiers) [35].

To design an *MCS*, an appropriate *fusion* method is required to optimally combine the individual classifier outputs to deter-mine the final decision (classification). Heterogeneous or homogeneous modeling backgrounds can be integrated (e.g., *LR*, *SVM*, and *random forest* (*RF*)) to exploit the strengths of each individual classifier and to overcome the limitations of an optimal local solution developed by an individual classifier. More-over, high diversity helps by decreasing the classifier output correlation [36, 37] and providing better options to explore different decision combination schemes. The most common *fusion rules* are majority, simple majority, and unanimous voting [15].

**Figure 5:**
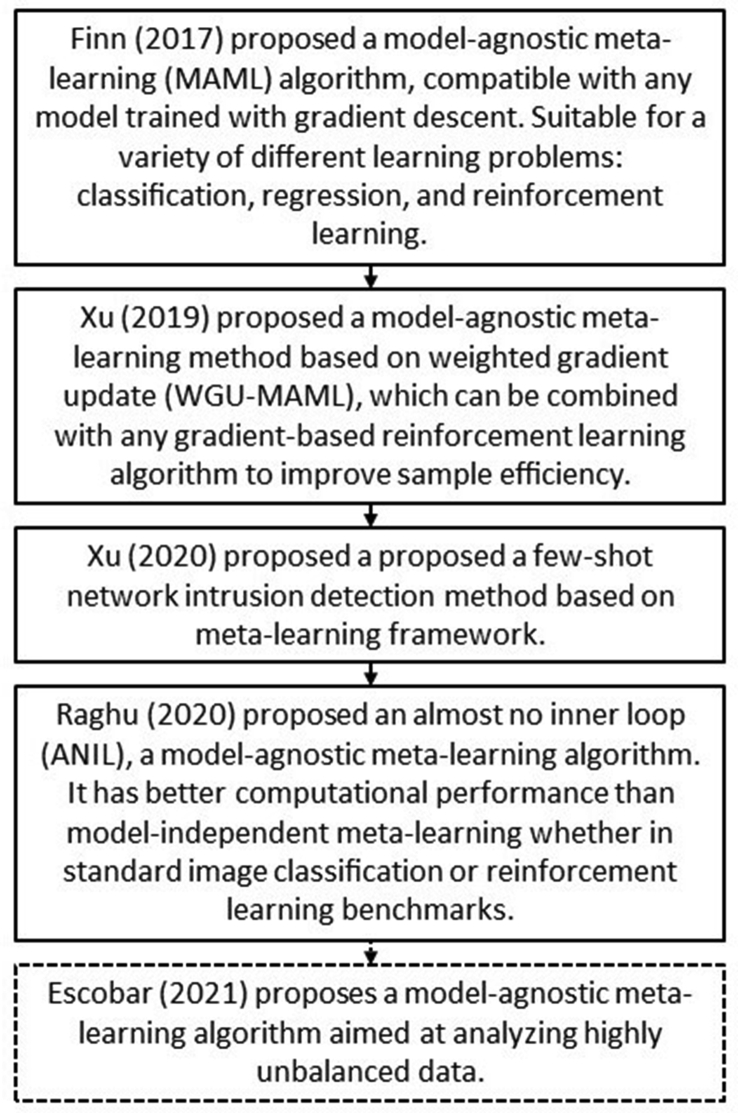
Recent developments of *meta-learning* algorithms.

*Meta-learning* is a broad topic in *AI* [20, 38]. This paradigm refers to an *MLA* that learns from the output of another *MLA* [39, 40]. A *meta-learning* algorithm is applied to dynamically learn or adjust the *fusion rule* of the *MCS* on the basis of the characteristics of the data at hand. It replaces static *fusion rules* or human involvement to manually evaluate different *fusion rules*. Figure 5 describes recent developments of *meta-learning* algorithms [41, 42, 43, 44]. A brief comment is included for each of them to briefly describe their diverse agendas.

### 3.2 List of MLA

To address the coverage optimization problem [22], a list of eight diverse and complementary *MLAs* is considered: *LR*, *SVM* [45], including *radial basis function* (*RBF*) kernels [46], *Naive Bayes* (*NB*) [47], *k-nearest neighbors* (*KNN*) [48], *artificial neural network* (*ANN*)^2^ [49], *RF* [31], and *random undersampling boosting* (*RUS-Boost*) algorithms (see Table 4). The proposed list includes margin- and probability-based, linear and non-linear [49], parametric and non-parametric [50], stable and unstable [51], and generative and discriminative [52] algorithms^3^. These approaches can be used to solve a wide spectrum of binary classification problems.

**Table 4:** Characteristics of the *MLA*.

Index	*MLA*	Linear	Nonlinear	Parametric	Nonparametric	Stable	Unstable	Gen	Dis
1	SVM	✓		✓		✓			✓
2	LR	✓		✓		✓			✓
3	NB		✓∗	✓		✓		✓	
4	KNN		✓		✓	✓			✓
5	ANN		✓	✓∗∗			✓		✓
6	SVM(RBF)		✓	✓	✓	✓			✓
7	RF		✓		✓	✓			✓
8	RUSBoost		✓		✓	✓			✓

∗with numeric features, ∗∗with a set of parameters of fixed size. Gen: Generative, Dis: Discriminative.

The first seven *MLAs* were initially proposed in [23]. They include the *RF* algorithm, a bagging approach. The original list is complemented with a boosting method. Because rare quality event detection is one of the primary applications of *PMQ* [10], *RUSBoost* [53] was selected as a boosting algorithm. *RUSBoost* is a combination of *random undersampling* and *AdaBoost* [54]. *Random undersampling* is applied to the majority class to balance the ratio between minority and majority classes, following which *AdaBoost* is applied to the balanced-subset to build a model.

The primary causes of error (i.e., misclassifications) are noise, bias, and variance. *Ensemble learning* (e.g., bagging and boosting) tends to produce a more reliable classification than a single classifier, and therefore minimizes these sources of errors. Although these methods are designed to improve the stability and robustness to noise, they have slightly different agendas for solving the bias–variance tradeoff [55]. In general, boosting tends to reduce the bias problem. Bagging may solve the over-fitting (variance) problem while boosting can increase it. Including both types of *ensembles* may be a good idea to effectively solve the bias–variance trade-off. Once a set of classifiers has been defined and trained, the next natural step is to rank them on the basis of a fitness function.

### 3.3 Maximum Probability of Correct Decision (MPCD)

*Maximum Probability of Correct Decision* (*MPCD*) is the fitness function to be optimized. It is a probability-based measure of classification performance, based on the α and β errors. It effectively analyzes highly/ultra-unbalanced data structures [11, 56] because its score essentially describes the ability of the classifier to detect the minority class, e.g., defective items. One has

(4) MPCD = (1 − α) (1 − β) ∈ [0, 1]

where a higher score indicates better classification performance. *MPCD* = 1 describes the perfect separation of classes, whereas *MPCD* = 0 describes either an α = 1 (all good called bad) or β = 1 (all bad called good)^4^. Once the classifiers have been ranked and the top performer identified, the next intuitive challenge is to determine whether two or more classifiers can be combined to improve the predictive benchmark of the top performer.

## 4 Classification optimization

A *meta-learning* algorithm (*meta-classifier*^5^) is presented. Its primary goal is to determine the *optimal fusion scheme* for *PMQ-O*. It generates a search space based on the set of classifiers and determines the *optimal fusion rule*.

**Figure 6:**
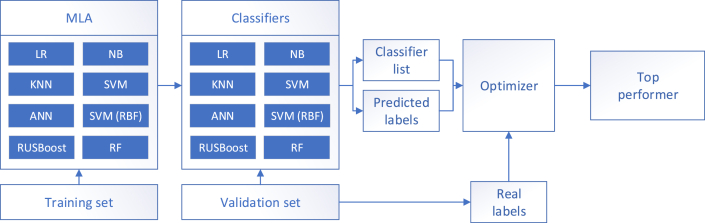
Top performer.

**Figure 7:**
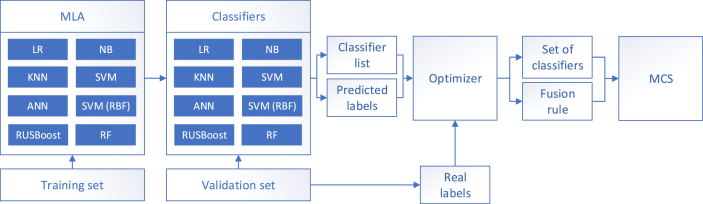
Multiple classifier system.

Because manufacturing systems tend to be time dependent, the data set should be split after a time-ordered hold-out vali-dation scheme (training and testing sets). The eight *MLAs* described in Table 4 are applied to the training set (e.g., first 70% samples) to create a set of classifiers. Thereafter, they are applied to the testing set (e.g., remaining 30 % samples). The *Real Labels* (*RLs*), *Predicted Labels* (*PLs*), and classifier list are the inputs of the optimizer that searches for a better solution (i.e., optimized prediction performance) with two possible outcomes: (1) top performer, a single classifier, or an *ensemble* (*RF* or *RUSBoost*); this situation occurs when no classifier fusion surpasses the benchmark set by the top performer (see Figures 6 and 2) *MCS*, a set of classifiers (it may include only a few or all) with a *fusion rule* that improves the benchmark set by the top performer (see Figure 7). The top performer may or may not be included in the *MCS*.

### 4.1 Optimizer pseudo-code

**Figure 8:**
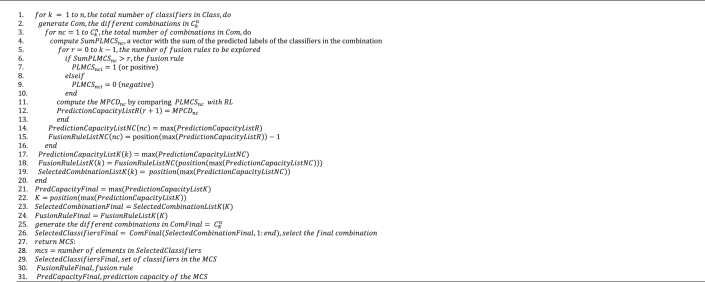
Optimizer pseudo-code.

The *PMQ-O* algorithm has three components (see Figure 8 and Table 5). It performs *n* iterations (*lines 1–20*), in each of which $C_{k}^{n}$ number of combinations are generated, where different combinations created at each iteration (*line 2*). Once the combinations have been defined, the algorithm performs *C* iterations to determine the best one (argmax{MPCD}) at each value of *k* (*lines 3–16*). To determine the best combination, a new vector is created, *SumLMCS* (see Eqn. 5), in which the values of the labels of the classifiers included in the combination are summed up (*line 4*). Thereafter, different fusion rules (*r*) are explored for each combination, starting with zero up to *k – 1*. The number of different *fusion rules* to be explored is equal to *k* (*lines 5–13*). To evaluate each *fusion rule*, the final *PL*, *LMCS* (see Eqn. 6), of the combination (*lines 6–10*) is used to compute the associated *MPCD* (*line 11*).

**Table 5:** Optimizer pseudo-code.

**(1) Inputs**
A set of classifiers and their associated predicted labels
• Class(*C*_*1*_*, C*_*2*_*, ..., C*_*n*_), a list of classifiers, n represents the number of classifiers
• *PL(pl*_*i*__*j*_*)*_*mxn*_, a matrix with predicted labels (*pl*_*i*__*j*_ *∈* {0, 1}) of the samples in the validation set, where m denotes the sample size, and the subscripts *i,j* are used to denote the *i*^*th*^ sample of the *j*^*th*^ classifier
• *RL(rl*_*i*_*)*_*m*_, a vector of size m with real labels (*rl*_*i*_ *∈* {0, 1})

**(2) Outputs**
*A Multiple Classifier System (MCS)*
• mcs the number of classifiers in the MCS
• SelectedClassifiersFinal, set of classifiers (*C*_*1*_*, .., C*_*mcs*_) in the *MCS* (it may include only the top performer)
• FusionRuleFinal, the fusion rule (zero is the fusion rule if only the top performer is included)
• PredCapacityFinal, estimated prediction ability

**(3) Initialization**
Define the algorithm's lists.
• Set *Com* as empty
• Set *SumLMCS* as empty
• Set *LMCS* as empty
• Set *MPCD* as empty
• Set *PredictionCapacityListR* as empty
• Set *PredictionCapacityListNC* as empty
• Set *FusionRuleListNC* as empty
• Set *PredictionCapacityListK* as empty
• Set *FusionRuleListK* as empty
• Set *SelectedCombinationListK* as empty

The *PredictionCapacityList* is used to store the associated *MPCD* to each different value of *r* for the combination under analysis (*line 12*). *PredictionCapacityListNC* and *FusionRuleListNC* lists are used to store the best *fusion rule r* of all the combinations developed at each value of *k* (*lines 14 and 15*). *PredictionCapacityListK*, *FusionRuleListK*, and *SelectedCombinationListK* lists are used to store the information (*MPCD*, *r*, *combination index* -*nc*- respectively) of the best combination at each value of *k* (*lines 17–19*). The final combination, *PredCapacityFinal*, is identified on the basis of the *MPCD* value (*line 21*). Because the dimensions of *Com* change at every iteration, the final combination must be generated again. At this point, all the information is available and is collected in *K* (*line 22*). *SelectedCombinationFinal* identifies the combination index (*line 23*) and the associated *fusion rule* in *FusionRuleFinal* (*line 24).* Using the values of *n* and *K*, the set of combinations in $C^{n}$are generated (*line 25)*, and the classifiers included in the index *SelectedCombinationFinal* are included in the *MCS* (*line 26*). Finally, the solution is reported (see Eqn. 7); *mcs*: the number of classifiers, *SelectedClassifiersFinal*, *FusionRuleFinal*, and *PredCapacityFinal* (*lines 27–31).*

(5) $$SumPLMCS=\sum L_{nc}$$

(6) $$PLMCS_{nci}=\left\{ \begin{array}{ll} 1 & \text{if ​}SumPLMCS_{nci}>r\Rightarrow i^{th}\text{item ​Pred. ​bad}\left(+\right)\\ 0 & \text{if ​}SumPLMCS_{nci}\leq r\Rightarrow i^{th}\text{item ​Pred. ​good}\left(-\right) \end{array}\right.$$

(7) $$MCS=\underset{nkncr}{\mathrm{argmax}}\left\{MPCD\right\}$$

The algorithm determines an optimal fusion scheme (i.e., number of classifiers, and fusion rule) for the *MCS*, where the search space is defined by the following:

(8) $$\sum_{k=1}^{n}k*C_{k}^{n}$$

## 5 Empirical studies

To gain insights into how the algorithm searches for *fusion rules* aimed at prediction optimization, a virtual case is validated.

### 5.1 Virtual case study

A sample size of 10 (*m* = *10)* with three bad(s) and seven good(s) is generated. The prediction of three classifiers *(n* = *3)* is input, and then the optimizer is applied to search for a *fusion rule* that enhances the prediction ability of the top performer. The *RLs* and *PLs* of each classifier, *Class*(*C*_1_, ..., *C*_3_), are presented in Table 6, and the prediction performance of each classifier is detailed in Table 7.

**Table 6:** Predicted labels of each classifier vs real labels, *k* = 1.

Sample	*PL*_*C*1_	*PL*_*C*2_	*PL*_*C*3_	*RL*
1	1	0	0	0
2	1	0	0	0
3	0	0	0	0
4	0	0	0	0
5	0	0	0	0
6	0	0	0	0
7	0	0	0	0
8	1	1	0	1
9	1	0	1	1
10	1	0	1	1

**Table 7:** Prediction performance (*k* = 1, *r* = 0).

*r* = 0	*C*_1_	*C*_2_	*C*_3_
*α*	0.2857	0	0
*β*	0	0.6667	0.3333
*MPCD*	0.7142	0.3333	0.6667

Tables 6 and 7 present the results of the different combinations in $C_{1}^{3}$. Because there is only one classifier in each combination (*k* = *1*), *r* = *0* is the only *fusion rule* to be evaluated, and *PL* = *SumPLMCS* = *PLMCS*. According to the prediction results, *C*_1_ is the top performer with *MPCD* = *0.7142*.

Now, the different combinations generated with *k* = *2* ($C_{2}^{3})$ are evaluated with respect to *r = 0* and *1*, where Eq. (5) is used to populate Table 8 (*sumPLMCS*). The classification rule described in Eq. (6) is then applied to define the *PLs* of each combination (PLMCS) (see Tables 9 and 10). Finally, Tables 11 and 12 present their associated predictive performances.

**Table 8:** SumPLMCS, sum of the values of the combinations created with *k* = 2.

Sample	$\sum PL_{C_{1}-C_{2}}$	$\sum PL_{C_{1}-C_{3}}$	$\sum PL_{C_{2}-C_{3}}$	*RL*
1	1	1	0	0
2	1	1	0	0
3	0	0	0	0
4	0	0	0	0
5	0	0	0	0
6	0	0	0	0
7	0	0	0	0
8	2	1	1	1
9	1	2	1	1
10	1	2	1	1

**Table 9:** PLMCS, predicted labels with *r* = 0.

Sample	$\sum PL_{C_{1}-C_{2}}$	$\sum PL_{C_{1}-C_{3}}$	$\sum PL_{C_{2}-C_{3}}$	*RL*
1	1	1	0	0
2	1	1	0	0
3	0	0	0	0
4	0	0	0	0
5	0	0	0	0
6	0	0	0	0
7	0	0	0	0
8	1	1	1	1
9	1	1	1	1
10	1	1	1	1

**Table 10:** PLMCS, predicted labels with r = 1.

Sample	$\sum PL_{C_{1}-C_{2}}$	$\sum PL_{C_{1}-C_{3}}$	$\sum PL_{C_{2}-C_{3}}$	*RL*
1	0	0	0	0
2	0	0	0	0
3	0	0	0	0
4	0	0	0	0
5	0	0	0	0
6	0	0	0	0
7	0	0	0	0
8	1	0	0	1
9	0	1	0	1
10	0	1	0	1

**Table 11:** Prediction performance (*k* = 2, *r* = 0).

*r* = 0	*C*_1_–*C*_2_	*C*_1_–*C*_3_	*C*_2_–*C*_3_
*α*	0.2857	0.2857	0
*β*	0	0	0
*MPCD*	0.7142	0.7142	1

**Table 12:** Prediction performance (k = 2; r = 1).

*r* = 1	*C*_1_–*C*_2_	*C*_1_–*C*_3_	*C*_2_–*C*_3_
*α*	0	0	0
*β*	0.6667	0.3333	1
*MPCD*	0.3333	0.6667	0

As per Table 11, the combination of classifiers *C*_2_ and *C*_3_ with a *fusion rule r* = *0* perfectly separates the data (*MPCD* = *1*) and surpasses the benchmark set by classifier *C*_1_ (*MPCD* = *0.7142*). Finally, *k* = *3* is evaluated. Because it is just one combination with the three classifiers, three rules are assessed, *r* = *0, 1,* and *2*. Following the same structure, the results are presented in Tables 13, 14, 15, 16, 17, 18, and 19.

**Table 13:** SumPLMCS, sum of the values of the combination created with k = 3.

Sample	*∑PLC*_1_*– C*_2_*– C*_3_	*RL*
1	1	0
2	1	0
3	0	0
4	0	0
5	0	0
6	0	0
7	0	0
8	2	1
9	2	1
10	2	1

**Table 14:** PLMCS, predicted labels with *r* = 0.

Sample	*∑PLC*_1_*– C*_2_*– C*_3_	*RL*
1	1	0
2	1	0
3	0	0
4	0	0
5	0	0
6	0	0
7	0	0
8	1	1
9	1	1
10	1	1

**Table 15:** Prediction performance (*k* = 3, *r* = 0).

*r* = 0	*C*_1_–*C*_2_ – *C*_3_
*α*	0.2857
*β*	0
*MPCD*	0.7142

**Table 16:** PLMCS, predicted labels with *r* = 1.

Sample	*∑PLC*_1_*– C*_2_*– C*_3_	*RL*
1	0	0
2	0	0
3	0	0
4	0	0
5	0	0
6	0	0
7	0	0
8	1	1
9	1	1
10	1	1

**Table 17:** Prediction performance (k = 3; r = 1).

*r* = 1	*C*_1_–*C*_2_ – *C*_3_
*α*	0
*β*	0
*MPCD*	1

**Table 18:** PLMCS, predicted labels with *r* = 2.

Sample	*∑PLC*_1_*– C*_2_*– C*_3_	*RL*
1	0	0
2	0	0
3	0	0
4	0	0
5	0	0
6	0	0
7	0	0
8	0	1
9	0	1
10	0	1

**Table 19:** Prediction performance (*k* = 3, *r* = 2).

*r* = 2	*C*_1_–*C*_2_ – *C*_3_
*α*	0
*β*	1
*MPCD*	0

As per Table 17, the combination of classifiers *C*_1_, *C*_2_, and *C*_3_ with a *fusion rule r* = *1* perfectly separates the data (*MPCD* = *1*). Finally, a comparative analysis combining the three classifiers with the three most common *fusion rules* (i.e., majority, unanimous-zero, and unanimous-one) and *PLs* are presented in Table 20. The classification performance is summarized in Table 21.

**Table 20:** Predicted labels for comparative analysis.

Sample	*PMQ-O*	Majority	Unanimous		RL
			zero	one	
1	0	0	1	0	0
2	0	0	1	0	0
3	0	0	0	0	0
4	0	0	0	0	0
5	0	0	0	0	0
6	0	0	0	0	0
7	0	0	0	0	0
8	1	1	1	0	1
9	1	1	1	0	1
10	1	1	1	0	1

**Table 21:** Prediction performance of the comparative analysis.

	*PMQ-O*	Majority	Unanimous	
			zero	one
*α*	0	0	0.2857	0
*β*	0	0	0	1
*MPCD*	1	1	0.7142	0

According to Table 21, although the majority vote resulted in perfect separation, this is not always the case [23]. However, as shown in this study, unanimous-zero (i.e., *r* = *0*) tends to be a good rule for rare event detection when the data set is highly/ultra-unbalanced. This *fusion rule*, however, tends to increase the *α* error. The unanimous-one rule (i.e., *r* = *n*) is on the other extreme; it tends to fail to detect or significantly increase the *β* error.

Static *fusion rules*, such as majority or unanimous voting, often times do not determine the optimal manner to combine the decisions of the classifiers because they do not eliminate highly correlated or spurious classifiers [36, 37] that degrade the generalization performance. However, the *meta-learning* algorithm presented only includes classifiers that optimize prediction. This is demonstrated in the following subsection.

### 5.2 Real case studies

To exhibit the performance of *PMQ-O*, six highly/ultra-unbalanced data sets (five publicly available) were analyzed using the eight *MLAs* (see Table 4). The name and relevant information for each data set are presented in Table 22, [23, 57]. The prediction performance on the testing set of each classifier is summarized in Tables 23 and 24 presents the information of the *MCS*.

**Table 22:** Data sets information.

Data set	Description	Features	Training set	Validation set	Ratio (overall)
1	UMW	54	18,495 (20)	12,236 (9)	0.09††
2	Statlog (class 1)	36	4,435 (1,072)	2,000 (461)	23.82†
3	Occupancy Detection	5	6,587 (173)	1,791 (98)	3.23†
4	HTRU2	8	12,000 (1,484)	5,898 (155)	9.15†
5	Sensorless Drive	48	44,000/14,509	4,000/1,319	9.09†
6	Credit Card Fraud	29	200,000 (385)	84,807 (107)	0.17††

†highly unbalanced ††ultra unbalanced. In parentheses the count of the positive class.

**Table 23:** Prediction ability (testing set) of each classifier by data set.

MLA		Data set					
		1	2	3	4	5	6
SVM	α	0.001	0.015	0.029	0.010	0.017	0.040
	β	0.222	0.013	0.031	0.103	0.055	0.094
	M	0.777	0.972	0.941	0.888	0.929	0.8704
LR	α	0.010	0.030	0.033	0.022	0.136	0.000
	β	0.111	0.022	0.010	0.077	0.124	0.252
	M	0.88	0.949	0.958	0.902	0.758	0.748
NB	α	0.006	0.138	0.033	0.025	0.089	0.003
	β	0.222	0.135	0.010	0.090	0.049	0.187
	M	0.773	0.746	0.958	0.887	0.867	0.811
KNN	α	0.000	0.003	0.023	0.009	0.004	0.000
	β	0.778	0.024	0.316	0.271	0.021	0.262
	M	0.222	0.974	0.668	0.723	0.975	0.738
ANN	α	0.011	0.012	0.033	0.027	0.011	0.009
	β	0.111	0.035	0.010	0.052	0.017	0.150
	M	0.879	0.954	0.958	0.92	0.972	0.843
SVM RBF	α	0.001	0.016	0.015	0.011	0.003	0.000
	β	0.778	0.017	0.327	0.090	0.007	0.402
	M	0.222	0.967	0.663	0.899	0.99	0.598
RF	α	0.000	0.014	0.026	0.008	0.003	0.000
	β	0.889	0.050	0.174	0.168	0.019	0.280
	M	0.111	0.937	0.805	0.826	0.978	0.720
RUST Boost	α	0.006	0.287	0.032	0.019	0.079	0.038
	β	0.222	0.089	0.000	0.142	0.030	0.150
	M	0.773	0.649	0.968	0.842	0.893	0.818

Top performer in bold, M means MPCD.

**Table 24:** PMQ-O results by data set.

Data set	Classifier(s)	*α*	*β*	*MPCD*	Fusion rule
1	LR,ANN	0.0076	0.1111	0.8821	1
2	SVM,SVM(RBF),ANN,KNN	0.0136	0.0065	0.9799	1
3	RUSBoost	0.0319	0	0.9681	0
4	ANN	0.0273	0.0516	0.9225	0
5	SVM(RBF),KNN,RF	3.790e-04	0.0030	0.9966	1
6	SVM,LR,ANN	0.0436	0.0841	0.8760	0

In data set #1, the top performer is the *LR* algorithm with an estimated *MPCD* = *0.8799*, *α* = 0.0101, and *β* = 0.1111 (see Table 23); this benchmark is improved (*MPCD* = *0.8821*) if *LR* is combined with the *ANN* with a *fusion rule* of 1. In this case, although the detection ability (*β*-error) is not improved, the *FP* (*α*-error) is reduced. In data set # 2, the benchmark set by the *KNN* (*MPCD* = *0.9736*) is higher (*MPCD* = *0.9799*) by the *MCSs* based on *SVM*, *SVM(RBF)*, *ANN*, and *KNN* algorithms with a *fusion rule* of 1. In this scenario, the detection ability is improved by 1.74% (from *β* = *0.0239* to *β* = *0.0065*) with a 1.1% loss of *α* (from *0.0026 to 0.0136*). In data sets #3 and #4, the benchmarks set by *RUSTBoost* and *ANN* are not improved by any decision combination. In data set #5, the benchmark set by *SVM*(*RBF*) (*MPCD* = *0.9901*) is improved (*MPCD* = *0.9966*) if the predictions of the *KNN* and *RF* are aggregated with a *fusion rule* of 1. In this case, detection is slightly improved, whereas the *α*-error remains essentially the same. Finally, in data set #6, the benchmark set by the *SVM* (*MPCD* = *0.8704*) is improved (*MPCD* = *0.8760*) by an *MCS* based on the *SVM LR* and *ANN* with a *fusion rule* of 1. In this case, the *α*-error increases by 0.37% (from *0.0399* to *0.0436*), whereas the *β*-error decreases by 0.94% (from *0.0935* to *0.0841*).

The proposed list of *MLAs* support *fusion strategy*. The top performer is a different classifier in each data set, and within each data set, classifiers tend to mislabel different examples. These results support the coverage optimization problem that recommends including a diverse and complementary list of *MLAs* to reduce dependency/redundancy.

Because the proposed algorithm performs an exhaustive search to determine an optimal solution, there are certain rare cases in which the solution is not unique. In this situation, it is recommended to generate a test set to evaluate the generalization ability of *MCSs* to a new unseen data set.

### 5.3 Limitations, recommendations, and managerial insights

*PMQ* uses real-time process data, which usually are in the form of signals, warranty data, direct quality observations, or coordinates. In these cases, each of the steps of the problem solving strategy are applied to solve the pattern classification problem. Moreover, as per a recent study, most of today's problems can be more effectively solved by simple *MLAs,* rather than *deep learning* [58]. *MLAs* simplify model interpretation and understandability, enabling engineering knowledge creation aimed at process redesign and improvement. However, there are multiple image-based applications aimed at visual inspection replacement (i.e., train a *deep neural network* to identify whether a screw is present in a transmission). In these types of applications, the seven steps do not apply and only a *deep neural network* structure can be used, such as a *convolutional*.

*PMQ-O* proposes a list of eight diverse *MLAs*. However, this list can be modified (*MLAs* can be added/eliminated/changed) on the basis of user preference/intuition. Adding (or removing) an *MLA* would increase (or decrease) the number of combinations to be evaluated. The *Six Sigma* approach or reaching of the maximum possible level in an organization is a business strategy, which summarized includes financial benefits, increased productivity, and a better customer satisfaction [59].

## 6 Industry 4.0 - technologies and sustainability

Implementing *Quality 4.0* practices indicates the maturity of an organization to pursue the excellence in performance [60]. This can only be achieved with digital transformation, making technology innovation and connectivity a priority. The collection of data needed for generation of insights and propositions of value in almost real-time is one of the main pillars in *I 4.0*. For quality improvement, industries have to enable the acquisition of data and measure current processes to have a starting point to know where the optimization needs to occur. Sensors and smart sensors let this information to be interpreted. According to [61], monitoring equipment and environmental conditions in a manufacture floor, allow for diagnosis and analysis of the processes, creating competitive advantages in companies implementing these practices. As a high amount of data is required for better decision-making, intelligent transducers or smart sensors are used for this purpose. They encompass both analog and digital sensors with their corresponding signal, a microprocessor and a communication protocol for data transfer [62]. These sensors can be arranged in a network for monitoring process and performance. The cross-sensor data validation allows a high reliability in both the sensors and the network, creating an identifiable and easy detection of problems [61].

As per [63], quality and the implementation of *Six Sigma* or the corresponding sigma level are directly related to manufacturing processes, although *Six Sigma* is a generic improvement methodology. Competitiveness is on a rise, and moving from *Four Sigma* to *Six Sigma* would mean improvements to be integrated into normal operations. The future benefits would include financial and technological development, standardization, and safety measure implementation. The variation in processes should decrease over time, and therefore, creating more robust systems, which would mean less maintenance and investments for upgrades. Certain *key performance indicators* (*KPIs*) in a successful implementation [64] include the following: (1) efficiency, (2) cost reduction, (3) time to de liver, (4) quality of the service, (5) customer satisfaction, (6) employee satisfaction, (7) reduced variation, and (8) financial benefits.

The following step from obtaining the necessary data is to communicate it and process it in almost real-time to prevent failures or detect defects. This *big data* approach and *ML* usage are the current standard for detecting gaps in the process. The business value of implementing real-time processing is the ability for instantaneous streaming of information and reaction [65] and this value has to prevail for a company to keep investing and develop new products and services.

The selection of the adequate solutions highly depend on the practitioners leading the engineering teams. As generations pass by, new challenges are found when training new technicians to handle new technologies and models. According to [66], in *I 4.0*, engineers must be able to understand mobile technology, embedded systems, sensors, network technology, machine-to-machine communication, robotics, *AI*, bionics, and safety competencies, along with other managerial skills such as leadership, financial analysis, and critical thinking. The value of these abilities will change the educational system to meet current and future global demands in the industry [67]. To close the gap between generations, education needs to transform and enhance the benefits that technology can bring to the industry. Some of the main changes in the workplace have been the flexibility to work without fixed hours or places, unconventional projects or opportunities, abroad programs, experimental jobs and the constant use of technology [68].

Even though training and the possession of technology are important, the value of an organization lays in its ability to sustain itself. The majority of companies are not prepared to tackle problems that could happen three or more years in the future, which can lead to poor implementation of *Quality 4.0* initiatives, as they only try to optimize operational efficiency immediately [69]. The steps in the path for sustainability, according to the North Highland Consulting Company, are: *(1)* To recognize transformation is required, *(2)* Envision the future and build the case, *(3)* Create the strategy and success measures, *(4)* Socialize and align, *(5)* Prioritize and organize, *(6)* Execute and implement. The implementation of these steps will ensure an excellent execution and a constant attention for customer and company needs.

## 7 Conclusions

In today's manufacturing world, most mature organizations have merged traditional quality-control methods to create high-conformance production environments. Process-monitoring charts are being applied to improve the process capability index, at the industrial benchmark of *sigma level four*, where only a few *DPMOs* are generated. Detecting these defects to move manufacturing processes to the *next sigma level* is one of the primary intellectual challenges posed to the application of *AI* to manufacturing processes.

*PMQ* is a *Quality 4.0* initiative aimed at defect detection, where rare quality event detection is its primary application. Detection is formulated as a binary classification problem. Because manufacturing-derived data sets for binary classification of quality tend to be highly/ultra-unbalanced, *PMQ-O*, an *MCS* with the capacity to effectively analyze these data structures, was developed.

*PMQ-O* is founded on a list of eight diverse *MLAs*, an *ad hoc* fitness function (measure of classification performance), and a novel meta-learning algorithm. This algorithm takes the prediction from a set of classifiers as input, and determines which classifiers are reliable and which are not, in predicting the final quality class. As per several publicly available data sets, the *meta-learning algorithm* outperforms widely used *fusion rules* because they do not adapt to the characteristics of the data.

Because *PMQ-O* improves prediction by determining the *optimal fusion rule* from a set of classifiers, this development is a step forward in the path of moving manufacturing processes to *the next sigma level*.

Future research along this path can focus on evaluating different fitness functions for *PMQ-O*. This would allow for the proposed method to generalize to other types of data structures and regression problems.

## Declarations

### Author contribution statement

Carlos A. Escobar: Conceived and designed the experiments; Performed the experiments; Analyzed and interpreted the data; Contributed reagents, materials, analysis tools or data; Wrote the paper.

Daniela Macias: Performed the experiments.

Ruben Morales-Menendez: Analyzed and interpreted the data; Contributed reagents, materials, analysis tools or data.

### Funding statement

This research did not receive any specific grant from funding agencies in the public, commercial, or not-for-profit sectors.

### Data availability statement

Data included in article.

### Declaration of interests statement

The authors declare no conflict of interest.

### Additional information

No additional information is available for this paper.

## Footnotes

1 *Quality 4.0* is the fourth wave in the quality movement (1. *Statistical Quality Control*, 2. *Total Quality Management*, 3. *Six Sigma*, 4. *Quality 4.0*). Its quality philosophy is built on the statistical and managerial foundations of the previous philosophies. It leverages *IBD* and *AI* to solve an entirely new range of intractable engineering problems. *Quality 4.0* is founded on a new paradigm based on empirical learning, empirical knowledge discovery, and real-time data generation, collection, and analysis to enable smart decisions [13].

2 *ANN* can be shallow or deep, depending on the available type of data.

3 The authors acknowledge that some algorithms can change their taxonomy (e.g., from parametric to non-parametric) depending upon their definition.

4 Since *MPCD* is the fitness function, it is recommended to tune the hyperparameters of the *MLA* with respect to this metric too.

5 *Meta-learning* algorithm for classification tasks.
